# Quantitative evaluation of cellular internalization of polymeric nanoparticles within laryngeal cancer cells and immune cells for enhanced drug delivery

**DOI:** 10.1186/s11671-021-03498-y

**Published:** 2021-03-02

**Authors:** Li-Juan Ma, Ruichao Niu, Xi Wu, Jun Wu, En Zhou, Xu-Ping Xiao, Jie Chen

**Affiliations:** 1 Department of Otolaryngology Head/Neck Surgery, Hunan Provincial People’s Hospital, The First Affiliated Hospital of Hunan Normal University, Changsha, 410005 People’s Republic of China; 2 Department of Respiratory Medicine, Xiangya Hospital, Central South University, Hunan Province, Changsha, People’s Republic of China

**Keywords:** Cellular uptake, Co-culture, Cytotoxicity, Laryngeal cancer, Polymeric particles, Cell fusion

## Abstract

Clinical translation of poly (lactic-co-glycolic acid) (PLGA)-based nanomedicine is limited, partly because of the poor delivery efficiency resulting from non-specific phagocytosis by phagocytes. Understanding the nanoparticle interplay between cancer cells and immune cells remains largely elusive. In this study, a quantitative investigation on cellular internalization of fluorescent PLGA particles (100 nm, 500 nm, and 1 µm) against laryngeal carcinoma cells with or without monocytes/macrophages in monoculture or co-culture systems was first performed. PLGA particles at concentrations of 5–20 µg/mL show superior biocompatibility except for 500 nm and 1 µm PLGA particles at 20 µg/mL slightly reduce cell viability. Microscopic observation has discovered all three sizes of particles are effectively ingested by both cancer cells and macrophages; however, quantitative fluorescence examination has disclosed that the uptake index of cancer cells (mean intracellular particle fluorescence per cancer cell normalized to that of per macrophage) is substantially declined for all PLGA particles in co-cultures compared to that in monocultures (1.35–1.05, 1.50–0.59, and 1.4–0.47 for 100 nm, 500 nm, and 1 µm particles, respectively). Quantitative analysis using flow cytometry further confirmed the reduced uptake index of cancer cells in co-cultures, but higher particle counts per macrophage. It has also been found that the formation of multinucleated giant cells via the fusion of macrophages increased after PLGA treatment, which could be further exploited as a potential approach for tumor drug delivery. Overall, these findings provide new insights into the interaction of nanoparticle-immune-cancer cells, which may facilitate the application of PLGA-based nanocarriers for the treatment of laryngeal carcinoma.

## Introduction

Cancer is one of the leading causes of mortality worldwide with around 10 million new cases of death reported in 2018 [1]. Laryngeal carcinoma (cancerous cells arise from the larynx) is the second most common malignancy of head and neck squamous cell carcinomas (HNSCCs), accounting for about 180,000 new cases and 95,000 deaths in 2018 [2]. Currently, targeted drugs are developed as an optional treatment for challenges introduced by traditional therapies such as surgery, radiotherapy, and chemotherapy [3]. Scientific efforts have been expedited to improve drug targeting and efficacy and reduce unwanted side effects by developing, for example, novel drug nanocarriers (*i.e.*, microneedles), personalized anticancer drugs, and therapeutic antibody targeted delivery systems [4].

Nanocarriers have been widely utilized to load anticancer drugs such as cisplatin, paclitaxel, and docetaxel to improve their water solubility, bioavailability, and stability for improved drug delivery and efficacy [3, 4]. In general, nanoparticle (NP)-based drug delivery permits the delivery of a broad range of other substances (*e.g.*, proteins, antibodies, vaccines, and nucleic acids) to specific regions of the body in both animal models and patients [4, 5]. However, the so-called enhanced permeability and retention (EPR) effect has resulted in considerably varying targeting efficiency among different types of cancer [6]. Recent evidence suggests that deposition of nano-drug-carriers (here gold NPs) to tumors preferentially depends on the process of transcytosis [7], a type of biological transcellular transport, in which substances/NPs are transferred across the cells from one side to another including the courses of endocytosis, vesicular transfer, and exocytosis. It should, however, be noted that the contradictory outcomes regarding the mechanisms of targeted drug delivery emphasize of critical importance for understanding the basis of cellular interaction with nano-drugs or NPs utilizing multiple experimental strategies ranging from in vitro cell culture to ex vivo tissue culture and in vivo animal study.

Numerous nanocarriers like liposomes, albumin NPs (NPs), silica NPs, and poly(lactic-co-glycolic acid) (PLGA) have been clinically employed to treat different types of cancer including laryngeal carcinoma [4]. Polymeric-based NPs such as micelles and PLGA hold great potential in biomedical applications because of their versatile formulations of nano-drug through simple mixing or covalent conjugation, excellent ability of self-assembly, high capability of drug loading, and biocompatibility [8, 9]. For example, polyethylene glycol (PEG)-coated PLGA nanocarriers loaded with doxorubicin and indocyanine green enable synergistic chemo-photothermal therapy for breast cancer [10]. Also, both in vitro and in vivo studies have shown that cisplatin-loaded micelles exhibit excellent anticancer activity against HNSCC orthotopic tumors (*i.e.*, SAS-L1 and HSC-2) [11]. Although several polymeric-based nano-drug-carriers like PLGA and micelles have been approved for clinical use or are being evaluated in clinical trials [4, 6], many more polymeric nano-drugs are under preclinical investigation with different head and neck cancer cell lines and xenograft tumor models of animals [11–14].

Scientific evidence has highlighted the significance of host immunological responses to nano-drug-carriers, because once those NPs once enter the body, they become unavoidably recognized by the immune system. Macrophages are considered the first line of cellular host defense, specializing in the neutralization and clearing of allergens, microorganisms, and foreign particles (*e.g.*, nanocarriers) via phagocytosis and consequent priming of immune responses. Most of the novel-designed nanocarriers have failed to targeted delivery to specific diseased regions or tumors in vivo because of the efficient accumulation of NPs in the mononuclear phagocyte system (MPS), such as Kupffer cells in the liver and red pulp macrophages in the spleen [15]. Hence, understanding the mechanisms of cellular uptake of NPs by immune cells like monocytes and macrophages is crucial as it determines the lifetime of nanocarriers in relevant tissues and biological fluids. Emerging in vitro cell co-culture system has, therefore, gradually attracted increasing attention within the fields of nanomedicine and toxicology because of the increasing demand for achieving more meaningful results that can better reflect the in vivo condition [16]. Indeed, co-culture systems have been proven to exhibit a realistic situation in mimicking healthy and diseased tissue states [17] and have been reliably utilized in NP cellular uptake and drug absorption studies [18–21]. Utilization of co-culture cancer cell and immune cell models generally offers a suitable platform for probing the uptake routes and mechanisms of these nanomaterials into cells, which may facilitate the design of nanocarriers that are better targeted to cancerous cells while simultaneously reducing NP phagocytosis. Therefore, it is of essential importance to determine the uptake efficiency and fate of NPs in cancer cells in the presence of immune cells. Most nanocarriers have been designed to have diameters of 50–200 nm to harness the EPR effect and prolong blood circulation, while larger NPs (> 500 nm) are reported to be efficiently cleared by the MPS [6]. PLGA, an FDA approved nano-carrier, in different sizes (100, 500, and 1000 nm) was thus selected to investigate the uptake capabilities of UM-SCC-17A (a classical laryngeal squamous carcinoma cell line) [22] and THP-1 (a human acute monocytic cell line) cells. Several in vitro studies have applied PLGA nanocarriers to deliver drugs to kill HNSCC cancer cells in monocultures [12, 13, 23], this is the first study to probe the uptake efficiency and mechanism of NP uptake by immune cells and laryngeal cancer cells synchronously in a co-culture model by employing different sizes of PLGA, which might provide a fundamental basis for the development of novel safe-by-design nanomedicine for HNSCC therapy.

## Materials and methods

### Materials

Three commercialized PLGA particles (Sigma-Aldrich) with different sizes including 100 nm, 500 nm, and 1000 nm were used in this study. All the particles were loaded with green fluorophores with optical excitation and emission (ex/em) wavelengths of 460 nm and 500 nm. All the particles were received in a form of powder and were suspended in distilled water with a final concentration of 10 mg/mL for further use. Hydrodynamic diameters and zeta potentials of three particles were carried out by dynamic light scattering (DLS) conducted with a Malvern Zeta Sizer Nano instrument (Malvern Instruments Ltd., Malvern, UK). The stock suspensions of each particle were 1:100 diluted in 80 µl distilled water for DLS measurement. Following the indication from the manufacturer, 3 repeated measurements for each particle were obtained.

### Cell cultures of UM-SCC-17A and THP-1 and particle exposure

The human laryngeal carcinoma cell line UM-SCC-17A and human monocyte/macrophage cell line THP-1 purchased from Sigma-Aldrich, USA and Shanghai Hengya Biotechnology Company (Shanghai, China), respectively, were used to build the in vitro model in this study. UM-SCC-17A cells and THP-1 cells were cultured in either DMEM [22] or RPMI-1640 cell culture medium supplemented with 10% FBS (Gibco, Germany) and 1% Penicillin–Streptomycin solution (Gibco, Germany) at 37 °C with 5% CO2. THP-1 cells were exposed to 100 nM Phorbol 12-myristate 13-acetate (PMA) (Sigma, USA) solutions for 72 h before cell seeding to differentiate into macrophages. Both of the cells were passaged using 0.5% trypsin–EDTA every 3 days, and the cell morphology was checked every day to ensure the health of the cells.

Cells were seeded in 24-well plates at the density of 0.1 × 10^6^ cells/well for mono-cultured cells for WST-1 and LDH assay, or on sterile glass coverslip in 24-well plates for fluorescent microscopy. For the co-cultured model, UM-SCC-17A cells were first seeded in 24-well plates at a density of 50,000/well overnight and then added with 50,000/well THP-1 cells. Particles were suspended in 500 µl either respective cell-culture medium for mono-cultured UM-SCC-17A and THP-1 cells or 1:1 mixed cell-culture medium for co-cultured cells and exposed to cell samples for 24 h with final concentrations of 5, 10, and 20 µg/mL for WST-1 and LDH assay or 10 µg/mL for fluorescent microscopy.

For FACS measurement, cells were seed in 12-well plates in the same manner as described before at a density of 250,000 cells/well, or 125,000 cells/well each for the co-cultured model. Cells were grown in 1 mL cell-culture medium overnight and exposed to PLGA particles at a final concentration of 10 µg/mL for 24 h.

### Cell viability assay

Cell viability was determined by the cell proliferation reagent WST-1 kit (Roche, Germany) according to the instructions from the manufacturer. Briefly, the WST-1 solution was 1:10 diluted in either respective cell-culture medium for mono-culture UM-SCC-17A or THP-1, or 1:1 mixed cell-culture medium for co-cultured cells. The supernatant was drained off after the exposure, and cells were incubated with 500 µl working solution of WST-1 assay at 37 °C for 30 min. Samples were collected and centrifuged at 14,000 rpm for 10 min to remove the particles. The absorbance (OD value) of the solutions were measured under the wavelength of 450 nm using Infinite^®^F200 (Tecan, USA). The absorbance values were corrected by subtracting the value of a blank sample containing only the WST-1 working solution, and the relative cell viabilities were compared with the untreated control sample.

### Cell membrane leakage assay

Lactate dehydrogenase (LDH) release was measured using a commercial cytotoxicity detection kit (LDH) (Roche, Germany) to determine the cytotoxicity by PLGA particles. The supernatant of the cells was collected 24 h after the exposure, centrifuged at 14,000 rpm for 10 min, and 1:10 diluted in 200 µl complete cell-culture medium. The positive control was defined as the total release of LDH by incubating the cells with 0.2% Triton X-100 at 37 °C for 15 min, and 1:50 diluted in 200 µl complete cell-culture medium. Samples were incubated with 100 working solutions of LDH assay for 30 min at room temperature, and the reaction was stopped using 50 µl 1% HCl. The absorbance at the wavelength of 492 nm was measured using Infinite®F200 (Tecan, USA), and relative LDH concentrations were calculated according to the following equation

$${\text{Relative}}\;{\text{LDH}}\;{\text{concentration}} = \left( {{\text{sample}}\;{\text{OD}}{-}{\text{blank}}\;{\text{OD}}} \right)/\left( {{\text{positive}}\;{\text{control}}\;{\text{OD}}{-}{\text{blank}}\;{\text{OD}}} \right) \times 5 \times 100\% .$$

### Fluorescence microscopy

Cells were visualized by Hoechst staining for localization of the particles under the fluorescence microscope. Cells were washed with PBS for 3 times after 24 h exposure of the particles and incubated with 4% formaldehyde for 10 min at room temperature. After fixed with formaldehyde, cells were then incubated with 200 µl staining solution containing 1:1000 diluted *Hoechst* and 1% BSA in PBS for 30 min. Then, the coverslips were moved onto a glass slide upside-down and maintained with a drop of DAKO fluorescence anti-fade agent for visualization. Four optical channels were set with a fluorescence microscope, including bright filed for cell morphology, DAPI for cell nuclei, and GFP for particles. Exposure times of the particle channel for each fluorescent picture were recorded and used for homogenization of the fluorescence intensity across different particles, and intracellular particles were calculated by fluorescence intensity using randomly selected areas by ImageJ (https://imagej.nih.gov/ij/). The uptake index across different particles was compared between mono-cultured or co-cultured UM-SCC-17A cells. Briefly, the mean fluorescent intensity (MFI) of internalized particles was calculated in, e.g., 50 cells for each cell type, which was determined as the subtraction value between the total fluorescence intensity (*I*_total_) of a specific area and the autofluorescence (*I*_auto_) of the same-sized area in the particle-free region equation [24, 25]. The uptake index of cancer cells was determined by the MFI of UM-SCC-17A normalized to that of THP-1 cells in either mono-cultured or co-cultured model. The calculation was performed following the equation below:

$${\text{uptake}}\; {\text{index}} = \frac{{I_{{{\text{total1}}}} - I_{{{\text{auto1}}}} \left( {{\text{MFI}}_{{{\text{UM}} - {\text{SCC}} - 17A}} } \right)}}{{I_{{{\text{total2}}}} - I_{{{\text{auto2}}}} \left( {{\text{MFI}}_{{{\text{THP}} - 1}} } \right)}}$$

### Fluorescence-activated cell sorting (FACS)

The fluorescence-activated cell sorting (FACS) was performed to measure the uptake capability of three particles. Cells were washed with PBS for three times to remove the cell medium and dissociative particles 24 h after the exposure and incubated with 200 µl 0.5% trypsin–EDTA (Gibco, Germany) at 37 °C for 4 min to remove the cells from the plate. Then, the reaction was stopped by adding 2 ml complete cell culture medium, and the cell suspension was moved to a glass tube for FACS measurement and centrifuged for 5 min at 300 × G, 4 °C. The supernatant was discharged gently, and cells were suspended in 200 µl PBS and preserved on ice. Live cells were first gated from debris and dead cells using forward (FSC-A)-side (SSC-A) scatter. Then, the co-cultured cells were analyzed using APC channel versus FSC-A to separate UM-SCC-17A cells from macrophages based on the size of cells. A total of 30,000 cells were analyzed for each sample, and the mean fluorescence intensity for each cell was calculated and normalized cross different particles. Meanwhile, after 24 h incubation of particles with cells, the cell culture supernatant, washing buffer (three times washing for remove the residual particles attached to the cell surface), and cells (trypsinized digestion) were collected to measure the fluorescent intensity using a microplate reader (Infinite®F200, Tecan). By this approach, the percentage of particles-ingested by cells can be determined for each group, for example, about 30,000 particles exposed to a single cell in a 12 wells/plate (250,000 cells total) at a dosage of 5 µg of 100 nm PLGA particles, resulting to average 13,000 particles internalized by single cells which is about 43% of the applied dose delivered to the cells. This delivered percentage can be further used to calculate the particles counts in the co-cultured system for each type of cells in FACS.

### Quantification of cell fusion

Macrophages fusion to multinucleated giant cells (MGC) was defined as a giant cell morphologically containing two or more nuclei within a normal cytoplasm, which can be clearly and synchronically identified in bright field images and fluorescent images after staining as recorded in the literature [26]. The percentage of cell fusion was calculated by the number of MGC nuclei (manually counting) normalized to the total cell counts, which was determined with an automated approach in ImageJ (https://imagej.nih.gov/ij/).

### Statistical analysis

GraphPad V8.0 software (GraphPad Software Inc., San Diego, CA, USA) was used for statistical analysis and results visualization. Following one-way ANONA, either Holm−Sidak method or t-test was carried out for comparing multiple-group results or two-group results, respectively. All the experiments were performed with independent triplications, and data were presented as mean ± standard deviation (STD). Results with *p* value < 0.05 (*) and *p* < 0.01 (**) were considered significant.

## Results and discussion

### Characterization of poly(lactic-co-glycolic acid) particles

**Fig. 1:**
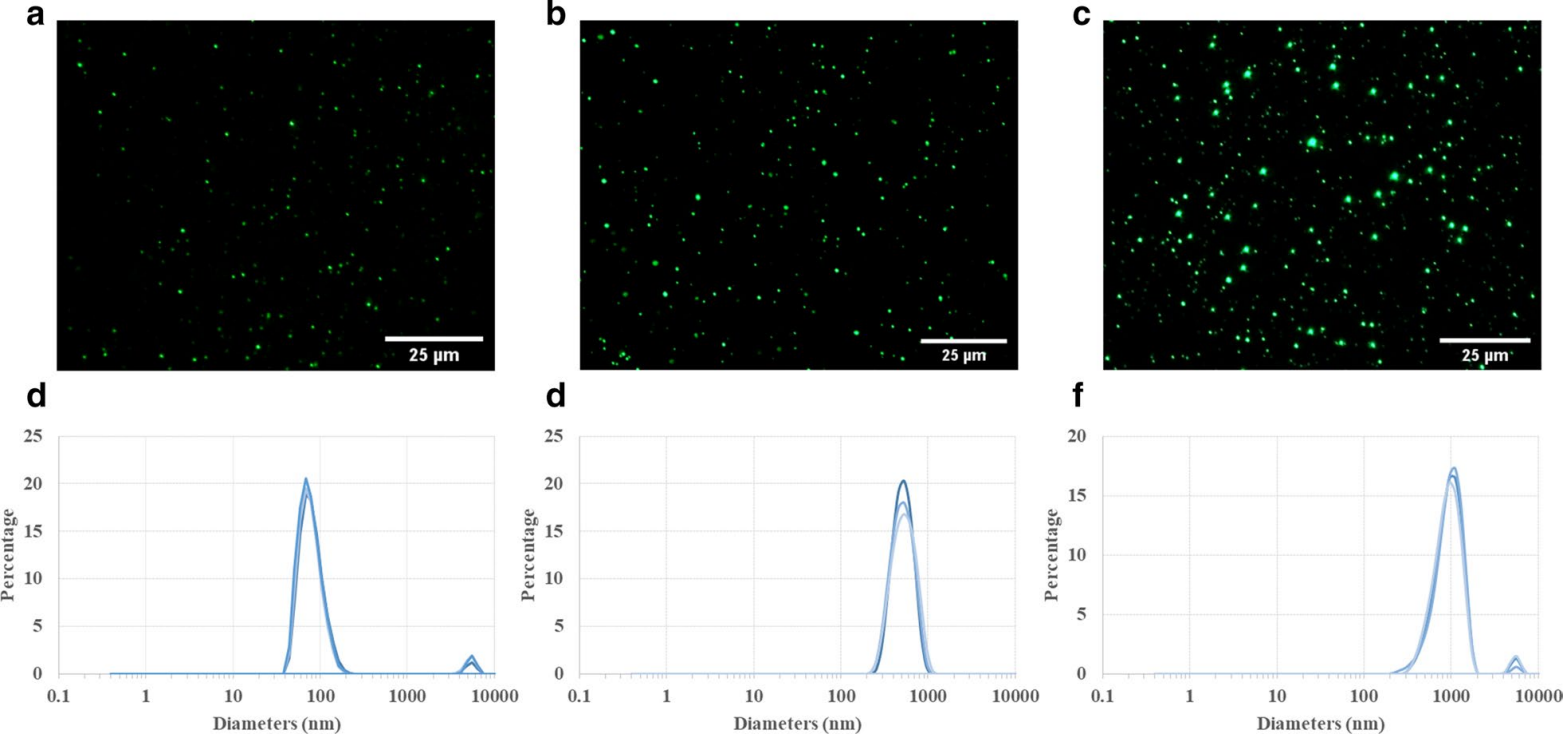
Characterization of PLGA particles. Fluorescence micrographs show the different sizes (**a** 100 nm, **b** 500 nm, and **c** 1 µm) of PLGA particles. Those particles can be detected under Olympus Optical Microscopy exhibiting relative uniform particle size distributions in water suspension. Dynamic light scattering (DLS) measurements display the size distribution weighted by volume for 100 nm (**d**), 500 (**e**), 1 µm (**f**) PLGA particles. Despite a small part of particle aggregation (smaller peaks: 3–4%) in 100 nm and 1 µm PLGA suspensions, the overall particle size distribution is quite homogeneous because of the narrow main peak

Fluorescence micrographs of PLGA particles (Fig. 1a,b,c) exhibited robust fluorescent intensity and no significant decreases in fluorescence signals over the course of 14 d after preparation (Additional file 1: S1), suggesting a relatively homogenous size distribution and high fluorescence stability. As denoted by the supplier, 100, 500, and 1000 nm PLGA particles showed the sizes of about 80.6 ± 19.3 nm, 542.6 ± 128.3 nm, and 951.9 ± 237.5 nm (Fig. 1d,e,f), respectively. According to zeta potential measurements, the average surface charges on the particles were found to be − 20.6 ± 5.3, − 17 ± 4.6, and − 16.5 ± 3.5 with poly dispersity index of 0.057, 0.056, and 0.062 for 100, 500, and 1000 nm particles, respectively, which the latter indicated their highly monodisperse standards. All particles were vortexed and then sonicated in a water bath for 5 min to largely eliminate the particle aggregation. However, small peaks corresponding to 100 and 1000 nm particles were observed with diameters of about 4–6 µm because of the inevitable particle aggregation in very small amounts (about 3–4%).

### Evaluation of cell viability and cytotoxicity

Due to their superb biocompatibility and biodegradability, PLGA particles have been approved by the FDA and European Medicine Agency for biomedical applications [27, 28]. PLGA particles have thus been currently used in the clinic and are widely applied in preclinical studies as nanocarriers delivering drugs to specific diseased regions or tumors. However, the targeting efficiency and therapeutic efficacy of PLGA particles are hindered, at least partially, by the immune system. For example, high particle phagocytosis by Kupffer cells in the liver greatly restricted the nano-drug-carriers from entering the tumor site [15]. Therefore, it is of vital importance to establish advanced in vitro models to study the interaction among cancer cells, immune cells and particles to better mimic the in vivo situation of drug delivery. UM-SCC-17A is a unique laryngeal squamous carcinoma cell line isolated from the primary larynx cancer specimen [29]. However, information regarding its in vitro cellular uptake efficiency in co-culture systems (*e.g.*, co-incubation of macrophages and cancer cells) is still insufficient, an issue that needs to be addressed to improve the prediction ability of in vivo responses. Moreover, it has been proven that particle size and surface coating play an important role in the delivery capability to solid tumors, diseased sites, and cancer cells in animal models and cell cultures [30–33]. Here, we thus applied three sizes of PLGA to UM-SCC-17A carcinoma cells to investigate the effects of particle size on cellular uptake and intracellular distribution in monoculture and co-culture systems.

**Fig. 2:**
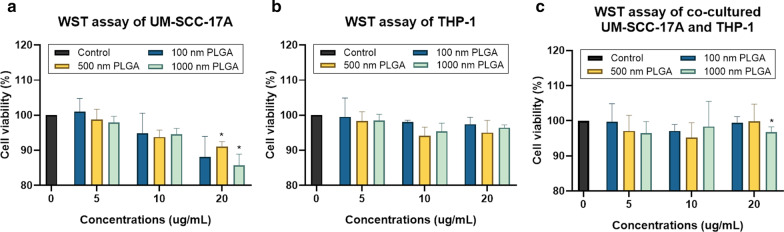
Determination of cell viability in monoculture and co-culture cells utilizing the WST assay. UM-SCC-17A (**a**) and THP-1 cells (**b**), and co-cultured UM-SCC-17A and THP-1 cells (**c**) were treated with various concentrations (5—20 µg/mL) of PLGA particles in three sizes

This study determined the cell viability using WST-1 (4-[3-(4-iodophenyl)-2-(4-nitrophenyl)-2H-5-tetrazolio]-1,3-benzene disulfonate) method for monoculture THP-1 and UM-SCC-17A cells, as well as a co-culture of both types of cells. Despite no obvious cell death was occurred in groups treated with 100 and 500 nm PLGA at all tested concentrations, 500 nm and 1 µm PLGA particles at the highest concentration (20 µg/mL) significantly reduced the cell viability in monoculture UM-SCC-17A cells (Fig. 2a). As expected, the THP-1 cell viability was not significantly affected after 24 h incubation with all three types of PLGA at concentrations of 5–20 µg/mL (≥ 95% viability in comparison with untreated cells, whose viability is considered as 100%, Fig. 2b). Identical to the results of monoculture UM-SCC-17A cells, cell viability in the co-culture system was not altered by the presence of 100 and 500 nm PLGA particles in three dosages used here, while it significantly declined in the group treated with 20 µg/mL 1 µm PLGA (Fig. 2c).

**Fig. 3:**
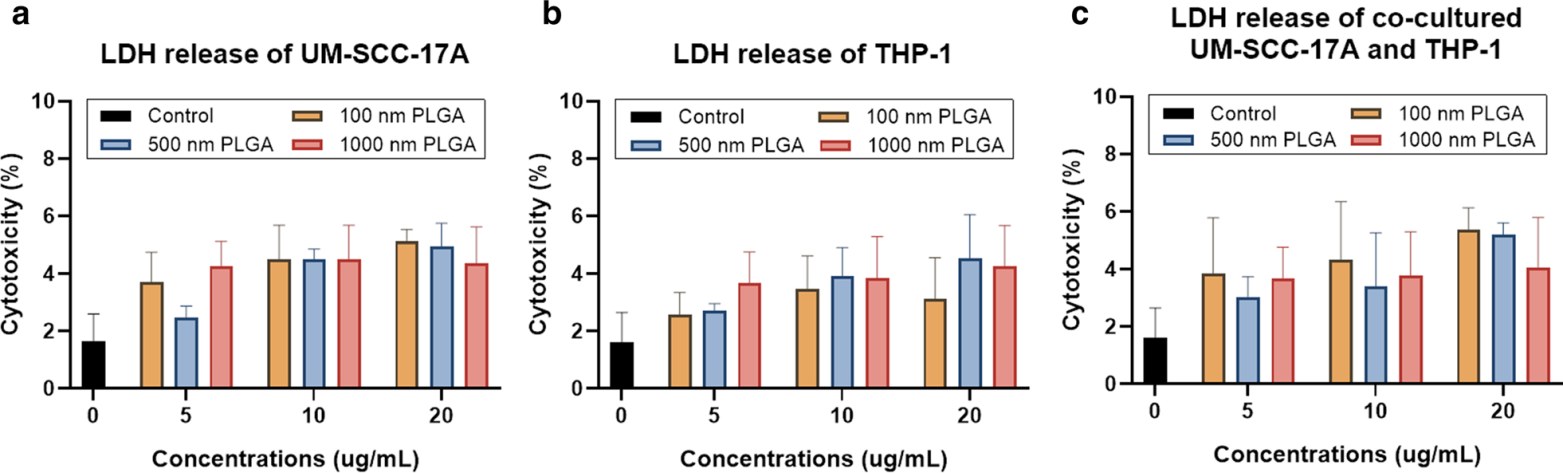
Determination of cytotoxicity to monoculture and co-culture cells using the LDH assay. UM-SCC-17A (**a**) and THP-1 cells (**b**), and co-cultured cells (**c**) were treated with PLGA particles. No significant cell death was observed after 24 h incubation of particles (with different sizes and concentrations used here) and cells in both monoculture and co-culture systems

Evaluation of particle cytotoxicity utilizing the LDH assay is to determine leakage of the cell membrane by measuring the amount of extracellular LDH [9]. The release of this cytoplasmic enzyme into the cell culture supernatant is characteristic of cellular membrane injury, leading to irreversible cell death. Despite the slight enhancement of LDH levels with higher dosages in monoculture UM-SCC-17A cells treated with various doses of 1 µm PLGA, no distinct cytotoxicity to the cells was observed even at the highest concentration of 20 µg/mL (Fig. 3a). Not surprising, all three sizes of PLGA used here at various dosages were unable to induce substantial LDH release into the supernatant, indicating the insignificant toxic effects to monoculture THP-1 cells, which is also highly consistent with the cell viability results described above (Fig. 3b). In the co-culture experiments, all the particles with different concentrations showed excellent biocompatibility toward both cells in terms of released LDH levels (Fig. 3c). In general, the broad particle size range, from 100 nm to 1 µm, that was used here covers the typical sizes of nanocarriers (50–200 nm), such as liposomes, micelles, dendrimers, polymers, and mini-cells. This range also covers sub-micron sized particles (500 nm particles can be still considered as NPs, *e.g.,* particles with sizes about 500 nm have same clearance pathways in the lung as those of NPs in 10–100 nm [34] and micron-sized 1 µm. None of these PLGA particles showed any obvious cytotoxicity to the THP-1 and/or UM-SCC-17A cells in monoculture and co-culture systems, except for the 500 nm and 1 µm PLGA particles at the highest concentration against UM-SCC-17A and co-culture cells (but more than 85% of cells are survived), indicating that they are favorable for applications in drug delivery systems.

### Capabilities of cellular uptake of PLGA in monoculture and co-culture systems

**Fig. 4:**
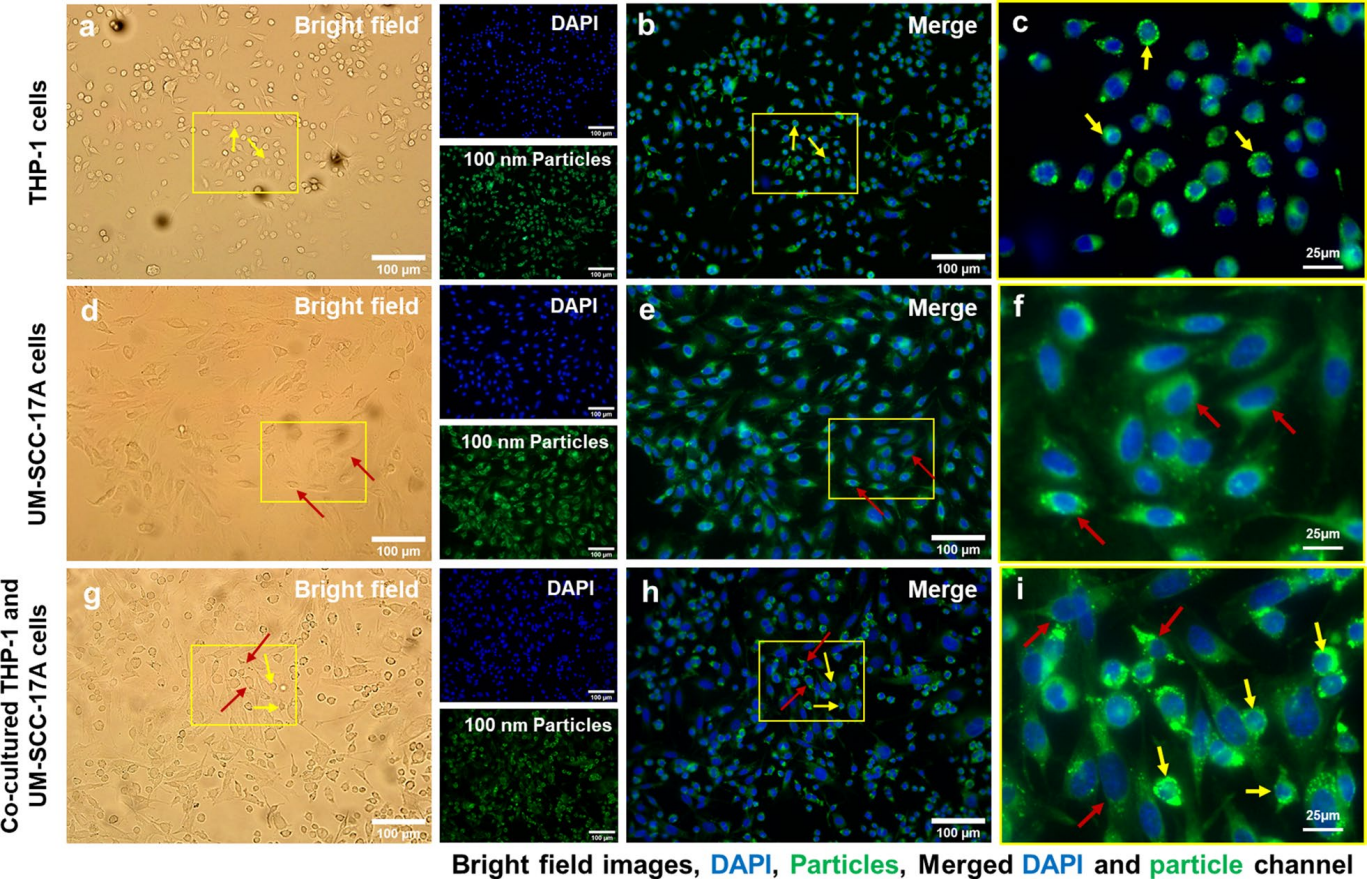
Microscopic examination of cellular internalization of 100 nm PLGA nanoparticles in monoculture and co-culture cells. Single type of cells or mixed cells was treated with 100 nm PLGA nanoparticles for 24 h at 37 °C at a concentration of 10 µg/mL. Cells were observed under bright-field (**a**, **d**, **g**) and fluorescent channels (b, e, and h) with particles observed at the green channel and cell nuclei in the blue channel after stained with Hoechst. Massive cellular uptake of 100 nm PLGA nanoparticles can be visualized in magnified images (**c**, **f**, **i**) for monoculture THP-1 macrophages (yellow arrows) and laryngeal cancer cells UM-SCC-17A (red arrows). In the co-culture system, 100 nm PLGA nanoparticles were still highly ingested by macrophages but less efficient for cancer cells compared to single cultured cells

**Fig. 5:**
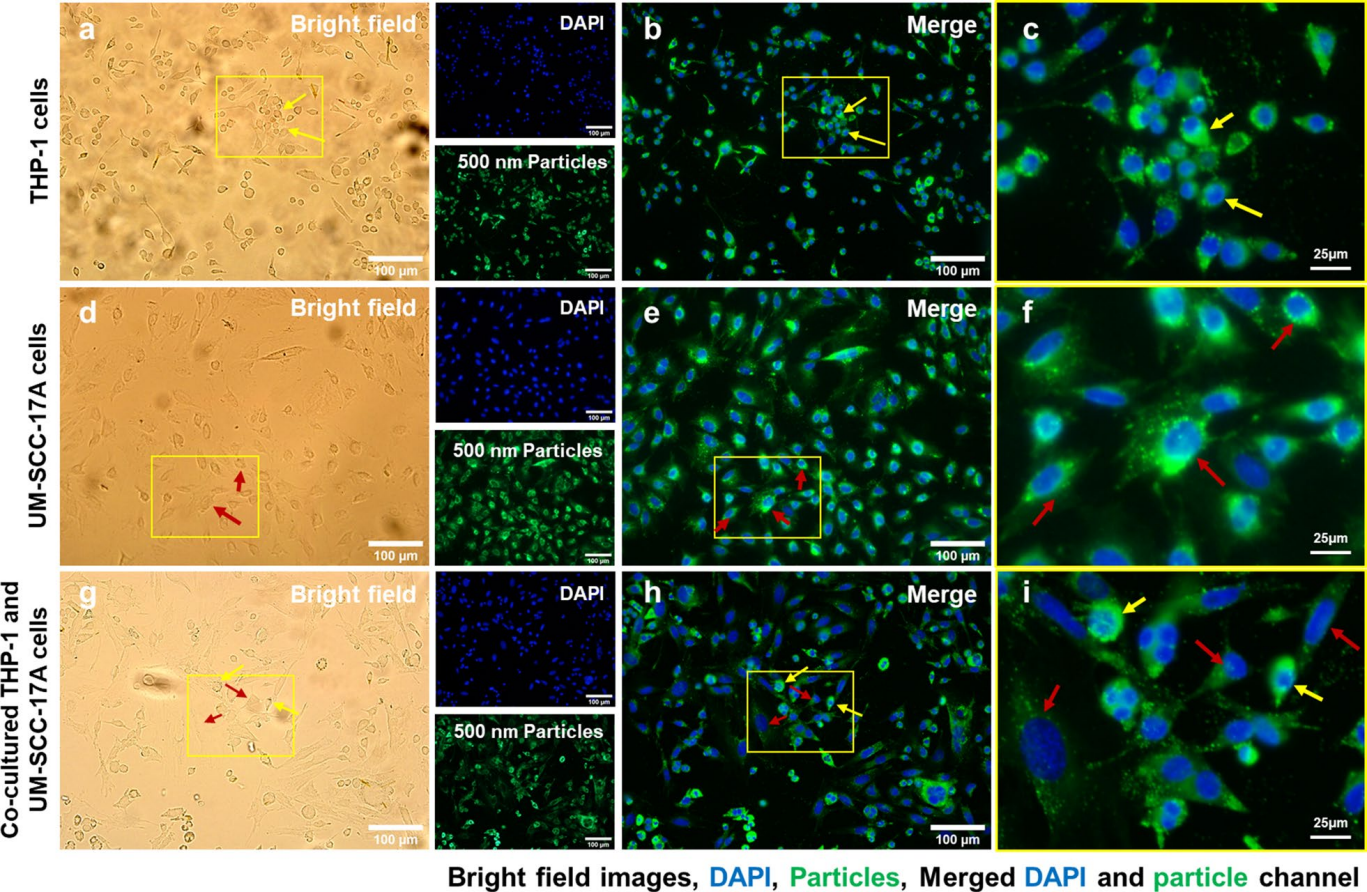
Microscopic examination of cellular internalization of 500 nm PLGA nanoparticles in monoculture and co-cultured cells. Single type of cells or mixed cells was treated with 500 nm PLGA nanoparticles for 24 h at 37 °C at a concentration of 10 µg/mL. Cells were observed under bright-field (**a**, **d**, **g**) and fluorescent channels (**b**, **e**, **h**) with particles observed at the green channel and cell nuclei in the blue channel after stained with Hoechst. Massive cellular ingestion of 500 nm PLGA nanoparticles can be visualized in magnified images (**c**, **f**, **i**) for monoculture THP-1 macrophages (yellow arrows) and laryngeal cancer cells UM-SCC-17A cells (red arrows). In the co-culture system, 500 nm PLGA nanoparticles were still highly uptake by macrophages, while cancer cells had inadequate particle internalization

**Fig. 6:**
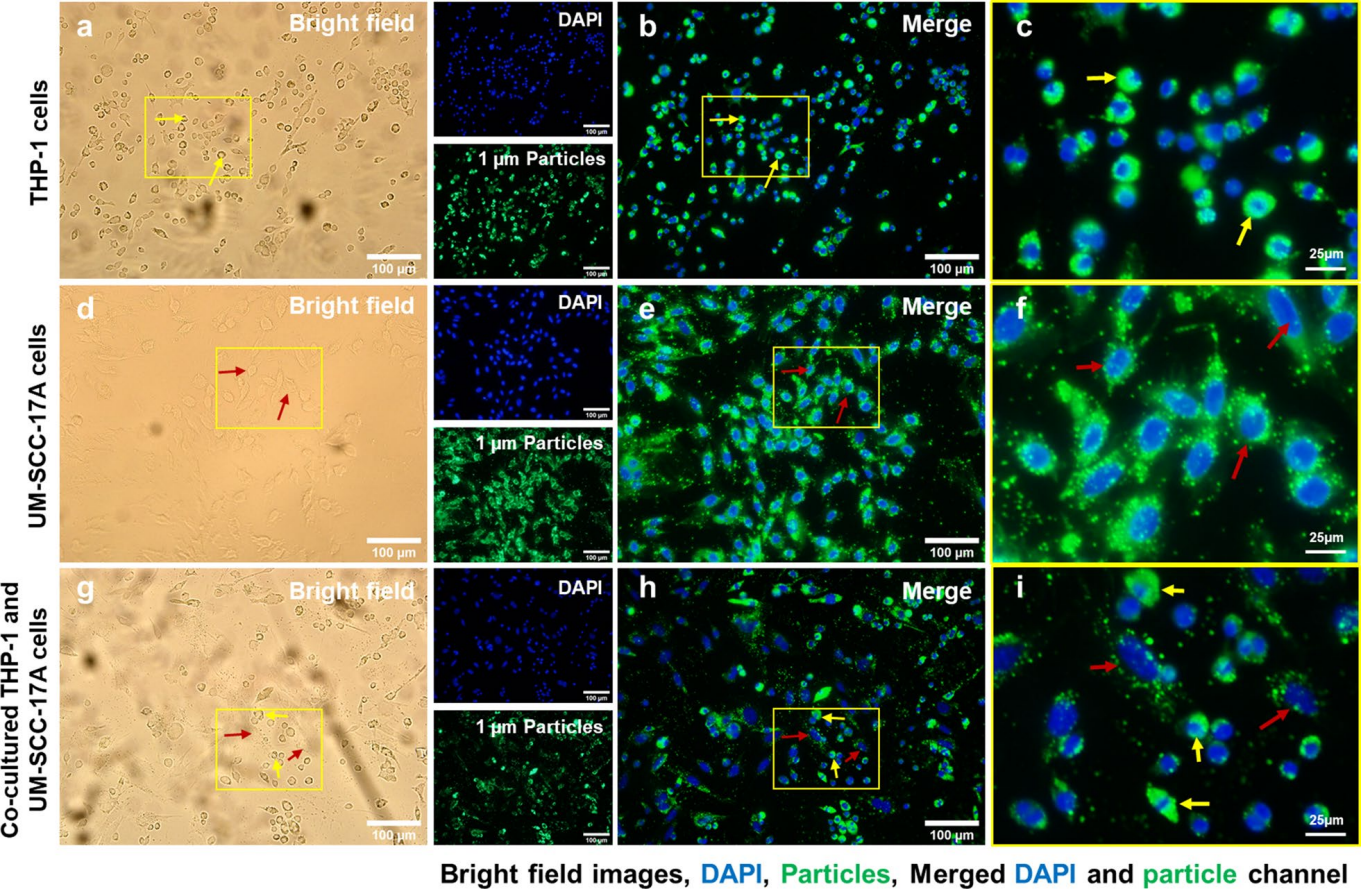
Microscopic examination of cellular internalization of 1 µm PLGA particles in monoculture and co-culture cells. Single type of cells or mixed cells was treated with 1 µm PLGA particles for 24 h at a concentration of 10 µg/mL. Cells were observed under bright-field (**a**, **d**, **g**) and fluorescent channels (**b**, **e**, **h**) with particles observed at the green channel and cell nuclei in the blue channel after stained with Hoechst. A large amount of particle uptake and tremendous accumulation of 1 µm PLGA particles can be visualized in magnified images (**c**, **f**, **i**) for monoculture THP-1 (yellow arrows) and UM-SCC-17A laryngeal cancer cells (red arrows). In the co-culture system, 1 µm PLGA particles were still efficiently uptake by macrophages, while cancer cells had insufficient particle ingestion

**Fig. 7:**
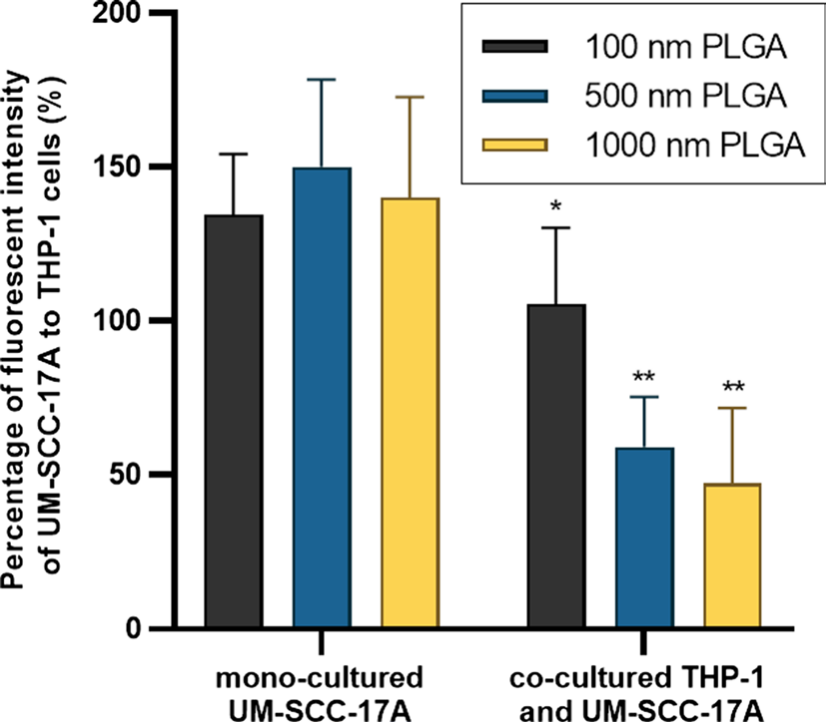
Quantitative analysis of PLGA particle internalization in UM-SCC-17A cells in monoculture and co-culture systems. The percentage of particle fluorescent intensity in UM-SCC-17A cells normalized to that in THP-1 cells for solely and mixed cultured cells

Figure 4 shows the cell morphology (Fig. 4a,d,g), cell nuclei and 100 nm NPs (Fig. 4b,e,h), and merged magnified images (Fig. 4c,f,i) in monoculture and co-culture systems. No particles were observed in the untreated cells of the control group (Additional file 1: S2). Massive large-sized 100 nm PLGA NPs agglomerates were observed in the cell cytoplasm of the macrophage monoculture cells (Fig. 4c). Meanwhile, monoculture UM-SCC-17A exhibited an excellent uptake capability of 100 nm PLGA, proven by the bright green fluorescence signals observed inside the cell membrane (Fig. 4f). To better illustrate the intracellular accumulation of PLGA particles in THP-1 or UM-SCC-17A cells and extracellular particles in the co-cultures, overlays of bright field images with fluorescence images were applied as in Additional file 1: S3). In co-culture system, both cell types are differentiable in terms of morphology and cell size in the bright field images, in which macrophages displayed a round shape and small size, whereas the cancer cells exhibited a large size and elongated shape. However, the cancer cells showed insufficient cellular ingestion due to the high uptake of 100 nm PLGA by macrophages in the co-cultured system. This is expected because macrophages are considered as an efficient and specific type of immune cells to perform the phagocytosis function for clearing foreign microorganisms, allergens, and particles. Similarly, Fig. 5 and Fig. 6 show qualitative analysis of cellular uptake ability of 500 nm and 1 µm PLGA particles in single-cell or mixed cell cultures, respectively. It is noteworthy that both cell morphologies were not altered after treatment with different sizes of PLGA particles at the concentration of 10 mg/mL used here (Fig. 4a,d,g, Fig. 5a,d,g, and Fig. 6a,d,g). 500 nm and 1 µm PLGA particles displayed stronger fluorescence intensity in sole UM-SCC-17A cells (Fig. 5e,f, and Fig. 6e,f) than that in sole THP-1 cells (Fig. 5b,c, and Fig. 6b,c). In co-culture incubations (Fig. 5h,i, and Fig. 6h,i), there are apparent signal decreases in cancer cells, in which the particle fluorescent intensity was found to be lower than that of the macrophages. Macrophages phagocytosed all types of particles rapidly and effectively so that cancer cells cannot ingest enough particles in co-culture system. To quantify the effect of phagocytosis on the uptake capacity by cancer cells for different sizes of PLGA particles, a semi-quantitative optical analysis was performed. Briefly, the particle fluorescence intensities were calculated on the basis of, for example, 50 individual particle-laden cells to achieve a mean value for both cell types, and the uptake index of cancer cells was determined by averaging the intracellular particle fluorescent intensity per cancer cell normalized to that of macrophages (Fig. 7). In monoculture, the cancer cell uptake index for 100 nm PLGA was found to be about 1.34 ± 0.19, suggesting that the cancer cells have ingested more particles than those ingested by macrophages in the single-cell culture (Fig. 7). This is not surprising because of the high level of viscosity of PLGA particles and the larger size of laryngeal cancer cells. Similarly, 500 nm and 1 µm PLGA were also greatly internalized by cancer cells with uptake indices of approximately 1.5 ± 0.25 and 1.4 ± 0.31, respectively, in monoculture. The uptake indices for large particles were substantially declined (0.59 ± 0.12 and 0.47 ± 0.1) in the presence of macrophages in co-culture systems. Meanwhile, identical cellular internalization capabilities for both cell types after 24 h incubation of 100 nm PLGA particles in the mixed cell culture. Overall, the results accumulated here suggested that the ingestion of particles by cancer cells and macrophages might depend on different routes of uptake pathway in single-cell culture and co-culture environment. Moreover, the presence of macrophages reduced the cellular internalization of PLGA particles by cancer cells particularly for large ones, a biological event that has been observed in in vivo nanomedicine drug delivery studies [3, 15].

### Quantification of cellular uptake by flow cytometry

**Fig. 8:**
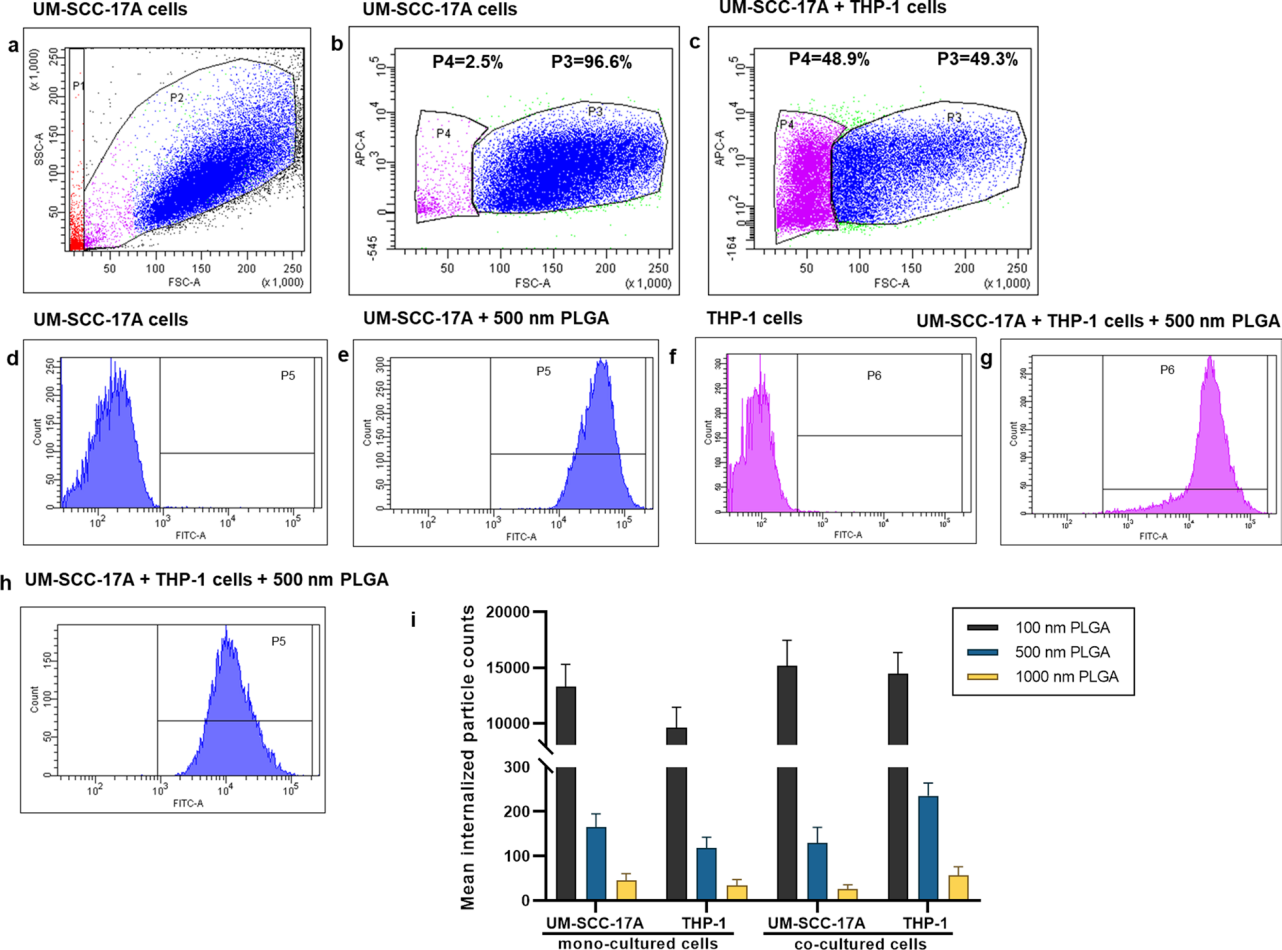
Particle uptake quantification in monoculture UM-SCC-17A cells and co-culture with THP-1 cells by flow cytometry. Grating strategy to identify respective cell populations in mixed cell culture (**a**–**h**). Gating is showing one representative experiment of cells exposure to 500 nm PLGA particles. With initial live gating in the y-axis with a side scatter (SSC-A) and x-axis with a forward scattering (FSC-A), P2 **a** were further gated with FSC-A versus APC-A to differentiate the THP-1 cells in P4 **c** from UM-SCC-17A cell population in P3 (monoculture cells (**b**) and mixed cells (**c**)). The cell population of P3 further displayed as counts versus FITC plots (P5) in non-exposed cells (**d**), monoculture cells (**e**), and co-cultured cells (**h**). Also, P6 is the counts versus FITC-A plot originated from P4 population in non-exposed cells (**g**) and co-cultured cells (**f**). Both types of cells efficiently ingested the 500 nm PLGA indicated by the solid histogram completed shifted to the right side of the *x*-axis, indicating particles are taken up by all exposed cells. Quantification of particle-laden numbers (**i**) in both types of cells for mono-culture and co-culture systems

Flow cytometry is widely used to determine of particle-cell interplay in a quantitative manner, for example, the size-dependent uptake of polystyrene particles with sizes ranging from 20 to 1000 nm by dendritic cells in vivo [35]. FACS was thus utilized for analysis of the percentage of NP-positive cells and particle number in those NP-positive cells in monoculture and co-culture systems. Then, average NP counts in a single cell were calculated by determining the total fluorescence intensity in particle-laden cells normalized to the fluorescence of total applied dose. For example, about 70% of 500 nm PLGA was deposited in the cancer cells, whereas the residual was kept in the supernatant of cell cultures (Additional file 1: S4). This fluorescence intensity measured by a Tecan reader was then compared to the average/total FACS signals (FITC signal values of P5 and P6 in Fig. 8,) to estimate the particle number in the co-culture system. As seeded in the co-cultured cells, about half of the cells were recognized as macrophages and the rest were considered as cancer cells. It was observed that approximately 13,000 of single 100 nm PLGA particles accumulated in cancer cells and 9700 particles in macrophages (Fig. 8i) in the monoculture, consistent with the above microscopic examinations. A much lower particle number (164 ± 30 and 45 ± 15) was ingested by monocultured cancer cells for 500 nm and 1 µm PLGA, with a slightly lower particle count in the respective macrophages (Fig. 8i). The number of particles ingested by single cells is in great agreement with the literature records [24]; for instance, an average 2500 of gold NPs coated with cetyltrimethylammonium bromide were deposited in epithelial cells, while PEG-modified gold NPs only had a few tens per cell [36]. In co-culture systems, unexpectedly, there is a slight increase of 100 nm particle number in cancer cells, despite a higher enhancement of NP internalization by macrophage. Comparing to the uptake index in the single-cell to mixed cell culture, it also reduces about 25% for 100 nm PLGA particles. The uptake indices were found to significantly decline with around 2.5 and 3 folds reduction in co-cultured system for 500 nm and 1 µm PLGA, respectively (Fig. 8i). Generally, it was found that the existence of macrophages largely affects the uptake ability of cancer cells especially for large particles, which is also in excellent agreement with the above observations.

Currently, different uptake pathways by cancer cells in ingesting particles with different sizes and surface modifications like clathrin-mediated endocytosis, caveolae-mediated endocytosis, clathrin- and caveolae-independent endocytosis, micropinocytosis, and macropinocytosis have been claimed in the literature [37]. For instance, it has been proved that iron oxide aggregates with a size of < 200 nm are taken up by MCF-7 cells through the clathrin-mediated endocytosis, whereas larger aggregates tend to be ingested via macropinocytosis [38]. Another study has demonstrated that 100 nm plain polystyrene particles tended to be taken up mainly through macropinocytosis, whereas the internalization of carboxylated polystyrene particles prefer to occur via the clathrin-mediated endocytic route [25]. Phagocytosis is a classical uptake pathway for immune cells such as neutrophils, dendritic cells, and most importantly monocytes/macrophages. The uptake pathways tightly depend on various parameters of drug delivery vesicles, *e.g.*, the particle size, chemical composition, surface modification, proteins in the culture environment, as well as cell type. Usually, multiple uptake pathways can be involved in particle ingestion such as the caveolae-mediated endocytosis, clathrin-mediated endocytosis, and macropinocytosis, which has been found to participate in cellular internalization of 300–400 nm chitosan NPs in human HeLa cells [39]. It has also been observed that 63 nm cholesterol-modified pullulan NPs enter into the human hepatocellular carcinoma (HepG2) cells via macropinocytosis and clathrin-mediated endocytosis [40]. Several previous studies showed that PLGA particle cell uptake involves different endocytic pathways, in which clathrin-independent endocytosis is the main route responsible for the internalization of PLGA in in vitro models [23, 24, 37]. Nevertheless, once they entered into the cells, PLGA particles applied here were highly potentially entrapped by the endo-lysosomal system consisting of early endosomes, recycling endosomes, late endosomes, and lysosomes [41]. It should be noted, however, that there is a lack of studies showing the cellular uptake mechanisms in co- or tri-cultured systems in vitro, a notion that has to be probe in the future.

### Induction of cell fusion by PLGA NPs

**Fig. 9:**
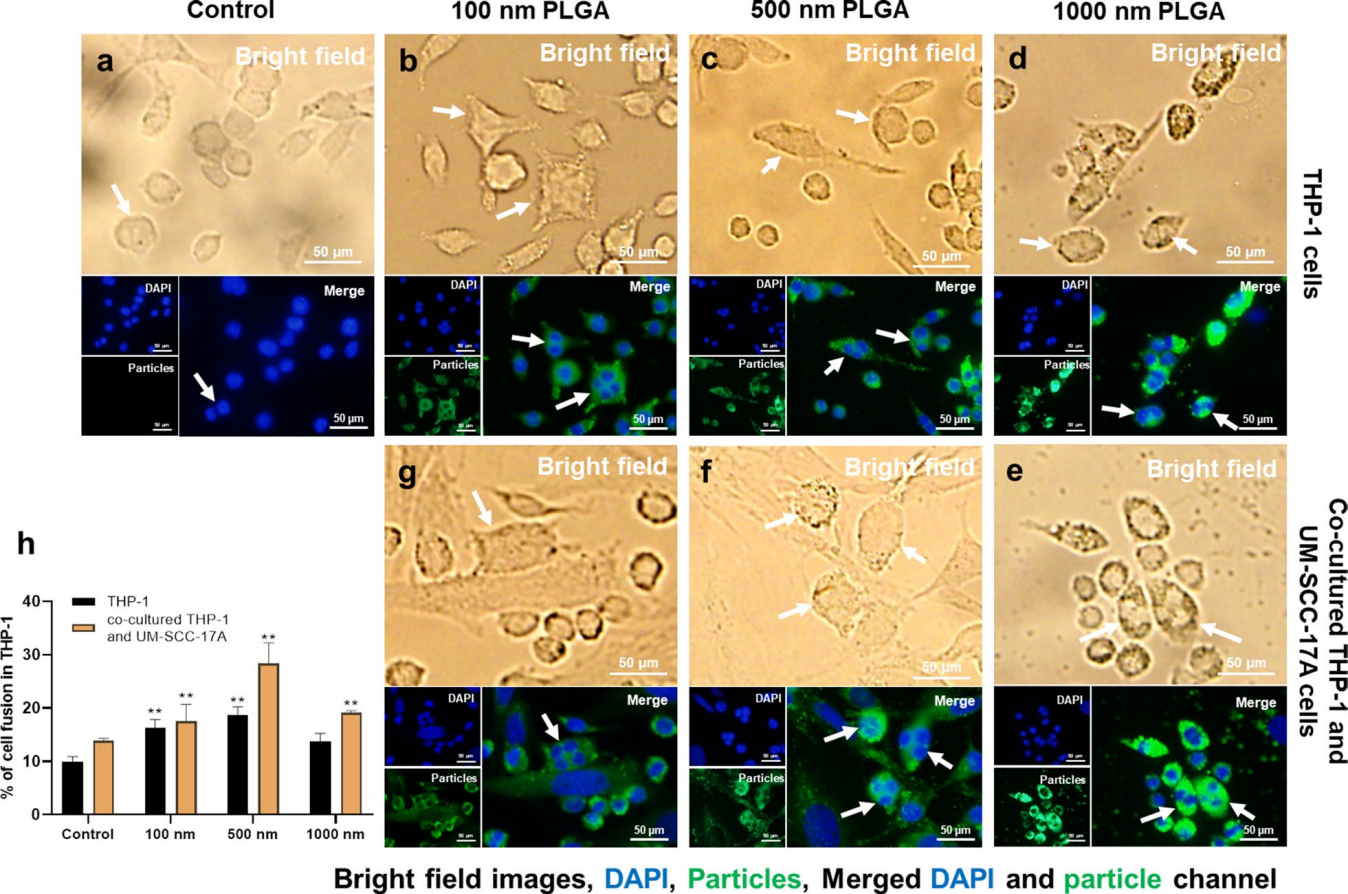
Visualization and quantification of THP-1 cell fusion in monoculture and co-culture systems. Cell fusion occurred in all groups including the control group without particle treatment for THP-1 cells (**a**). Monocultured (**b**, **c**) 100 nm and 500 nm NP-induced significant cell fusion compared to 1 µm PLGA group (**d**), which has a slightly higher level than that of the control group. In co-cultured systems, 100 nm (**g**) and 1 µm (**e**) PLGA had an identical percentage of cellular fusion, which is apparently smaller than that of 500 nm group (**f**). Quantification of cell fusion for all groups after 24 h incubation was displayed in **h**

Extensive scientific evidence has shown that the fusion of monocytes/macrophages into multinucleated giant cells (MGCs) occurred in a broad range of biological processes [42, 43]. Generally, implantation of biomaterials into the body causes a foreign body response characterized by the fusion of macrophages into MGCs and fibrotic encapsulation [44]. A wide type of human and murine primary cells like alveolar macrophages, splenic macrophages, microglia, bone-marrow-derived macrophages, and blood monocytes, as well as cell lines such as RAW264.7 and UG3 were also frequently observed to form MGCs in vitro [26, 45]. It is well-known that the macrophages form a fusogenic phenotype when they are unable to ingest foreign materials via phagocytosis because of the large size of particles or implants. So far, it is unclear whether in vitro macrophage cell fusion relates to the particle size in the range 100–1000 nm, which can be easily phagocytized. Here, the cell fusions were observed in all groups by microscopic examination (Fig. 9). The cellular fusion was confirmed only when multiple nuclei were found to share the same cytoplasm in both fluorescence images and bright-field images. Spontaneous formation of giant cells by THP-1 cells was occurred in the control group without particle treatment (Fig. 9a), with a fusion percentage of about 10% of the total cells (Fig. 9h), as reported in the literature [46]. Size-dependent cellular fusion was found for 100 and 500 nm PLGA particles, whereas 1 µm PLGA-induced insignificant enhancement of fusions in monoculture. This may be due to the small number of 1 µm PLGA particles exposed to cells (approximately 60 particles exposed to each cell at a concentration of 10 µg/mL). When the macrophages were co-cultured with cancer cells, the percentages of MGC formation were increased in all groups in comparison with the corresponding monoculture groups. In particular, there is a significant increment of cellular fusion for 1 µm PLGA (19%) in the mixed cell culture. The most prominent increase in fusion occurred in co-cultured 500 nm PLGA particles, which might be attributed to the large size of particles and sufficient particle numbers ingested per cell.

The molecular machinery involved in macrophage fusion has been widely probed, achieving substantial progress [43]. For example, the formation of macrophage fusion receptors CD47and CD44 together allowed mediating the process of macrophage fusion, and subsequent the differentiation of giant cells [42] and miR-223 delivery by a NP vesicle permitted attenuating it [47]. Hence, further study should focus on the determination of key molecules in regulating particle-induced macrophage fusion and the dominant receptors expressed in the giant cell membrane. Another important concern is the fate of MGCs. It is believed that MGCs fused with particles or stimuli might subsequently experience the process of apoptosis or necrosis [48]. This may lead to the release of undigested particles or other giant materials to the other cells in vitro or biological tissues in vivo, further inducing the long-term inflammatory response or granulomas. On the other hand, the macrophage fusion itself may be benefit for NP drug delivery, for example, in tumorous tissues, the re-release of drugs from macrophages to the cancer cells might represent an enhanced tumor killing ability. More importantly, future nanomedicine study should address how to exploit the application of macrophage fusion for nanocarrier drug delivery to improved disease diagnosis and therapy. This is because not only macrophages an abundant type of immune cells in the body with the excellent uptake ability of nano-drugs, but also they may be used in targeted gene delivery for repair of injured tissues.

## Conclusion

To the best of our knowledge, this study first reported the competitive cellular uptake of PLGA particles with sizes ranging from 100 nm to 1 µm in co-cultured UM-SCC-17A laryngeal cancer cells and THP-1 monocytes/macrophages. The data collected here proved that immune cells may alter/lower the particle internalization by cancer cells in vitro, which is similar to the previous findings in in vivo nanocarrier drug delivery studies. Size-dependent and cell culture-related macrophage cellular fusion caused by PLGA particles has also been demonstrated here. Future studies should probe the uptake mechanism in co-cultured systems and design novel approaches to achieve a higher uptake index for laryngeal cancer cells in the presence of phagocytes. Moreover, elaborate evaluation of intracellular trafficking and fate of nano-drug-carriers in co- or tri-cultured systems in 3D, which better mimic the in vivo conditions, is needed prior to the utilization of animal studies in vivo and even in clinical trials.

## Supplementary Information

**Additional file 1: Fig S1.** Fluorescent intensity of various PLGA particles over two weeks after preparation. **Fig S2**. No particles can be observed in the untreated cells of control group. **Fig S3**. Co-localization of bright field images (b) with fluorescent images (c) displays the intracellular accumulation of PLGA particles in THP-1 cells (red arrows) or UM-SCC-17A cells (white arrows) and extracellular particles (yellow arrows) in the co-cultures (d). **Fig S4**. Percentages of PLGA particles deposited in the cells (intracellular particles) normalized to the applied particles (the total dose) at 24 h after incubation.

## Data Availability

All data generated or analyzed during this study are included in this published article [and its supplementary information files].

## Abbreviations

PLGA: Poly(lactic-co-glycolic acid)
HNSCC: Head and neck squamous cell carcinoma
EPR: Enhanced permeability and retention
NPs: Nanoparticles
ICG: Indocyanine green
MPS: Mononuclear phagocyte system
LDH: Lactate dehydrogenase
FACS: Fluorescence-activated cell sorting
MGC: Multinucleated giant cells
DLS: Dynamic light scattering
