# Supplementary material for: Quantitative evaluation of cellular internalization of polymeric nanoparticles within laryngeal cancer cells and immune cells for enhanced drug delivery
Source: Nanoscale Res Lett. 2021 Mar 2;16:40. doi: 10.1186/s11671-021-03498-y (PMC7925719; doi:10.1186/s11671-021-03498-y)
Supplement: Supplementary file 1 — Additional file 1: Fig S1. Fluorescent intensity of various PLGA particles over two weeks after preparation. Fig S2. No particles can be observed in the untreated cells of control group. Fig S3. Co-localization of bright field images (b) with fluorescent images (c) displays the intracellular accumulation of PLGA particles in THP-1 cells (red arrows) or UM-SCC-17A cells (white arrows) and extracellular particles (yellow arrows) in the co-cultures (d). Fig S4. Percentages of PLGA particles deposited in the cells (intracellular particles) normalized to the applied particles (the total dose) at 24 h after incubation. [file 11671_2021_3498_MOESM1_ESM.pdf]

Supplementary information

Quantitative evaluation of cellular internalization of polymeric nanoparticles within laryngeal cancer cells and immune cells for enhanced drug delivery

Authors and affiliations:

Li-Juan Ma[1#]*, Ruichao Niu[2#], Xi Wu[1], Jun Wu[1], En Zhou[1], Xu-Ping Xiao[1], and Jie Chen[2]*

[1] Department of Otolaryngology Head/Neck Surgery, Hunan Provincial People's Hospital, The First Affiliated Hospital of Hunan Normal University, Changsha, 410005, P.R. China;

[2] Department of Respiratory Medicine, Xiangya Hospital, Central South University, Changsha, Hunan Province, P.R. China;

[#] These authors contributed equally to this work.

* Corresponding Author:

Li-Juan Ma, Department of Otolaryngology Head/Neck Surgery, Hunan Provincial People's Hospital, The First Affiliated Hospital of Hunan Normal University, Changsha, 410005, P.R. China;

Email: horserma@163.com;

Jie Chen, Department of Respiratory Medicine, Xiangya Hospital, Central South University, Changsha, Hunan Province, P.R. China;

Email: chenjie869@163.com;

Tel.: +86-7318-227-8012;

Fax: +86-7318-227-8019

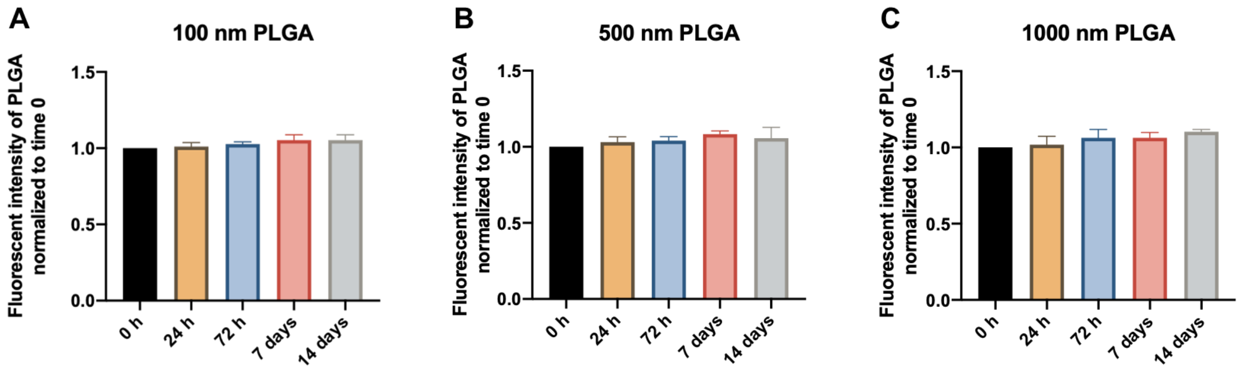

Figure S1: Fluorescent intensity of various PLGA particles over two weeks after preparation. All fluorescent signals were normalized to the initial signal at time 0 after preparation.

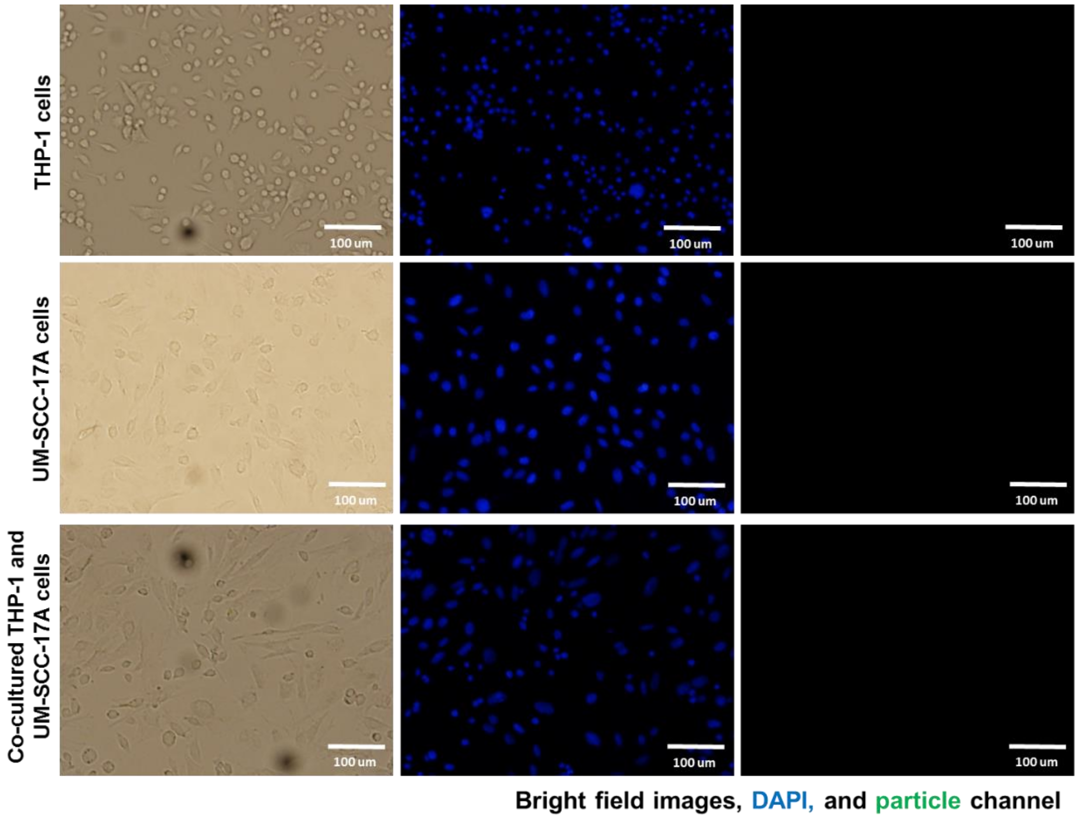

Figure S2: No particles can be observed in the untreated cells of control group.

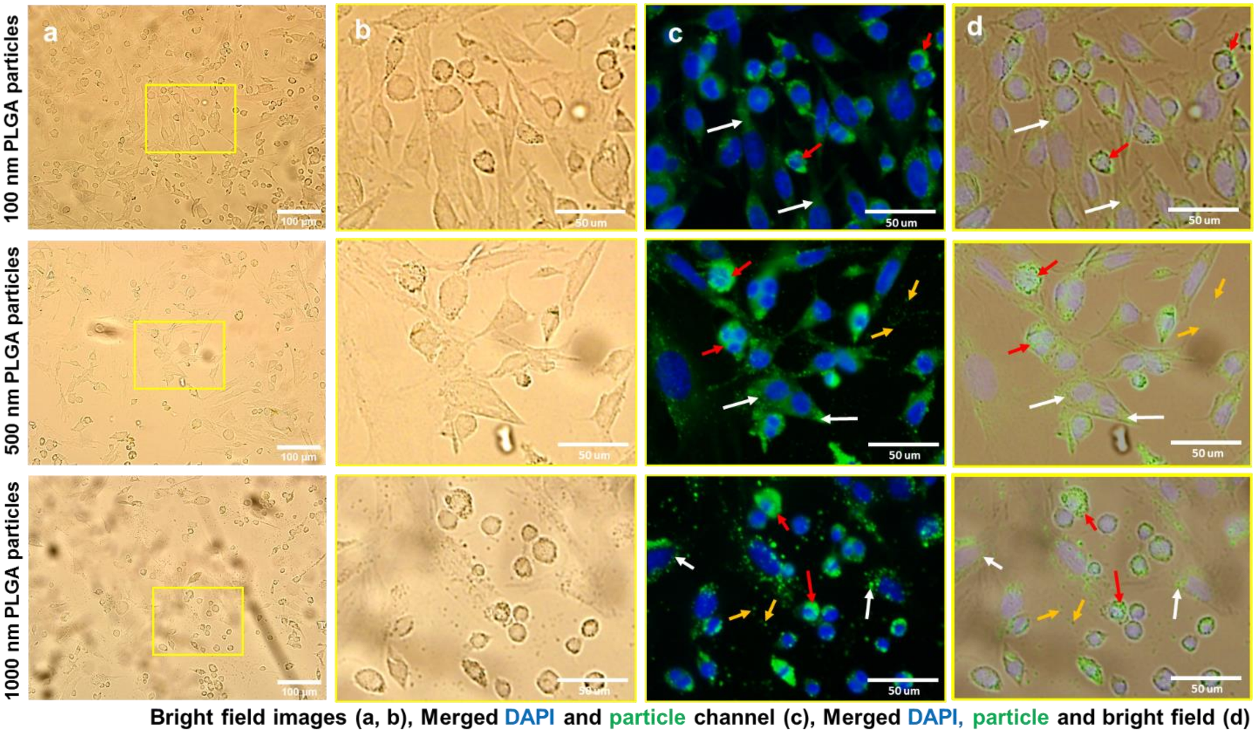

Figure S3: Co-localization of bright field images (b) with fluorescent images (c) displays the intracellular accumulation of PLGA particles in THP-1 cells (red arrows) or UM-SCC-17A cells (white arrows) and extracellular particles (yellow arrows) in the co-cultures (d).

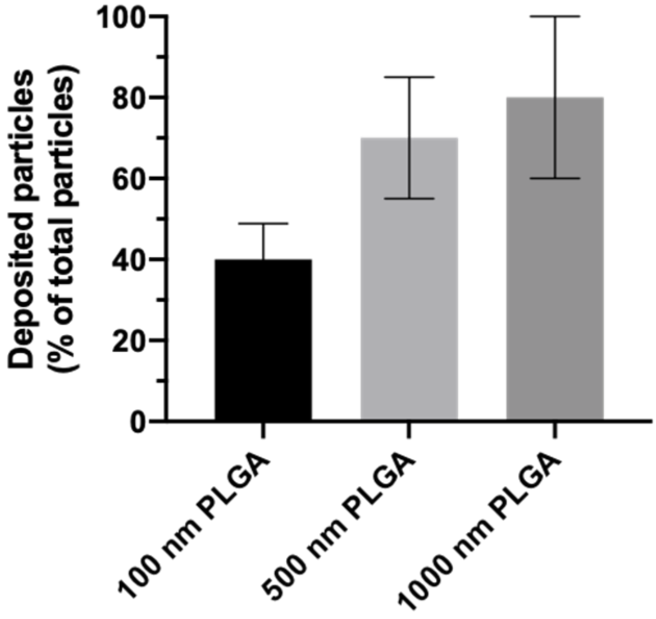

Figure S4: Percentages of PLGA particles deposited in the cells (intracellular particles) normalized to the applied particles (the total dose) at 24 h after incubation.
